# Supplementary material for: Evidence or Confidence: What Is Really Monitored during a Decision?
Source: Psychon Bull Rev. 2023 Mar 14;30(4):1360–79. doi: 10.3758/s13423-023-02255-9 (PMC10482769; doi:10.3758/s13423-023-02255-9)
Supplement: Supplementary file 1 — (PDF 137 kb) [file 13423_2023_2255_MOESM1_ESM.pdf]

Supplementary Material

for

Evidence or Confidence: What is really monitored during a decision?

Douglas G. Lee[1,2] Jean Daunizeau[3,4] Giovanni Pezzulo[1]

[1] Institute of Cognitive Sciences and Technologies, National Research Council, Rome, Italy

[2] Tel Aviv University, School of Psychological Sciences, Tel Aviv, Israel

[3] Paris Brain Institute (ICM), Paris, France

[4] Translational Neuromodeling Unit (TNU), ETH, Zurich, Switzerland

Simulation results related to the empirical hurdles for confidence models

In the main manuscript, we report a collection of results related to a set of so-called empirical hurdles that has been proposed for computational models of choice confidence to clear (Pleskac & Busemeyer, 2010). We showed that our current model, the confidence-based drift-diffusion model or cDDM can clear most of the hurdles with no issue. However, some of those results were not obvious without the aid of simulations beyond those reported in the main text. Here, we describe the relevant supplemental simulations and present the associated results that were summarized in the main Results section.

Hurdle 6

Average decision times are typically longer for inaccurate versus accurate responses. For this result, we used the identical simulated data as for all the primary results. As shown in Figure S1, across the full range of value difference (dV), average response times were longer for inconsistent (the subjective value analog of inaccurate) trials than for consistent (the subjective value analog of accurate) trials.

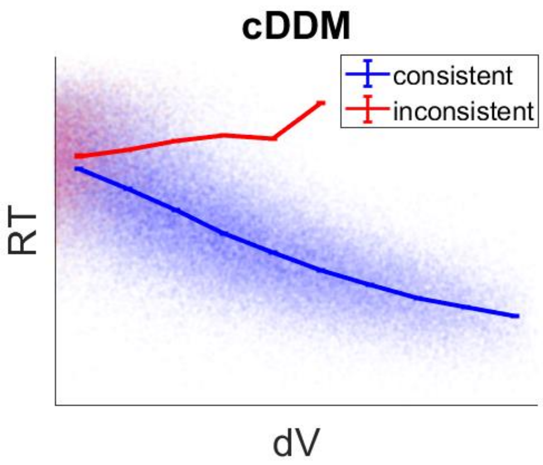

Figure S1: **Responses are slower on inconsistent versus consistent trials.** *Response time (RT) is generally longer for inconsistent trials than for consistent trials (where the chosen option aligns with value ratings). Each dot represents one trial; curves show the means across equally-spaced dV bins.*

Hurdle 7

Average decision times can be *shorter* for inaccurate versus accurate responses when choice difficulty is low and speedy responses are encouraged. For this result, we simulated ne data while setting the diffusion noise parameter higher than in the main analyses (12 versus 6). As shown in Figure S2, when considering only “easy” trials (high dV), average response times were shorter for inaccurate versus accurate trials.

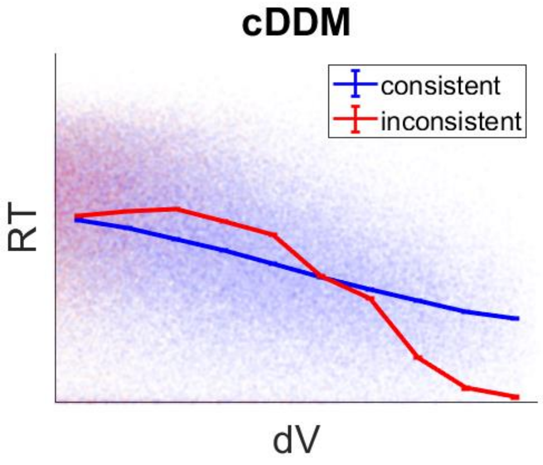

Figure S2: **Responses can be faster on inconsistent versus consistent easy trials.** *Response time (RT) can be shorter for inconsistent trials than for consistent trials, if the trials are easy*

(high dV) and if there is a lot of (diffusion) noise in the evidence sampling process. Each dot represents one trial; curves show the means across equally-spaced dV bins.

<u>Hurdle 8</u>

Under time pressure, the resolution of confidence increases: the gap between average confidence for accurate versus inaccurate responses increases. For this result, we simulated data while setting the response threshold to collapse more quickly, consistent with the idea that decision-makers will terminate deliberation more abruptly when concerned about taking too much time. As shown in Figure S3, the gap between confidence for consistent and inconsistent trials is larger in the speedy condition compared to the normal condition.

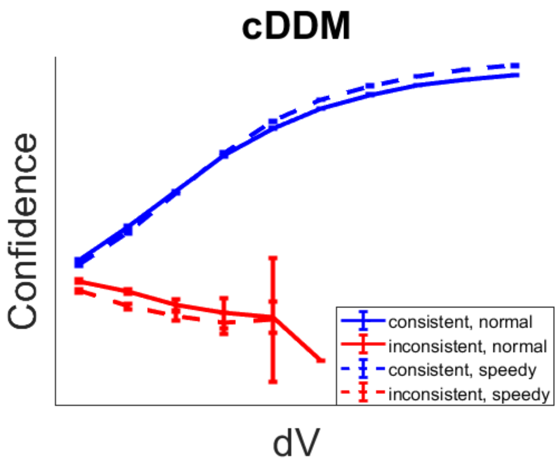

Figure S3: Confidence resolution increases with time pressure. *The gap between confidence on consistent versus inconsistent trials (blue and red curves, respectively) increases under conditions of time pressure or otherwise speedy choices (dashed curves) compared to normal free-response conditions (solid curves).*
